# Supplementary material for: Data driven network inference and longitudinal transcriptomics unveil dynamic regulation in Chronic Lymphocytic Leukaemia models
Source: NPJ Syst Biol Appl. 2026 Jan 15;12:24. doi: 10.1038/s41540-025-00645-4 (PMC12894745; doi:10.1038/s41540-025-00645-4)
Supplement: Supplementary file 1 — Supplementary Information [file 41540_2025_645_MOESM1_ESM.pdf]

Supplementary Material

Deconvolution analysis

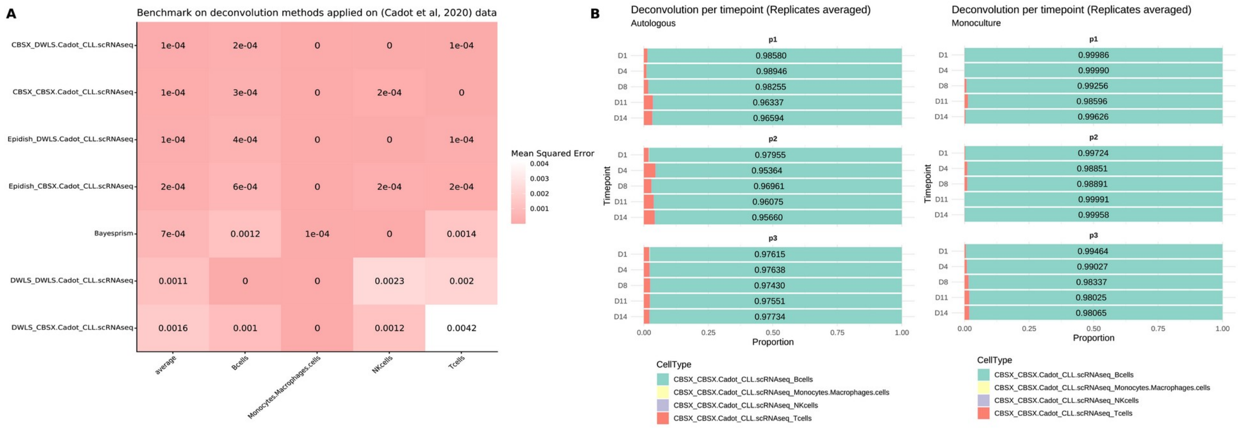

Fig. S.1: Deconvolution performed on autologous and monoculture, using single-cell RNA-seq-derived signatures. **A** Heatmap with performance of signatures, **B** Cell percentages in autologous culture and monoculture, using the custom signatures with CBSX. All the necessary scripts to reproduce the results can be found in the GitHub repository.

Microscopy images of the *in vitro* culture

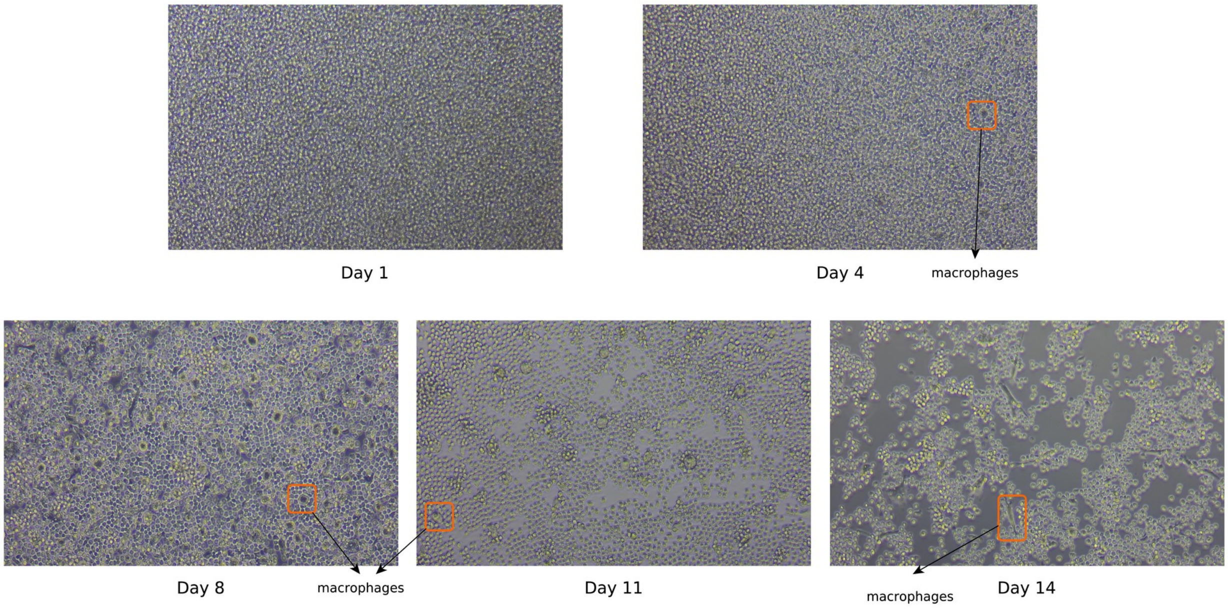

Fig. S.2: Microscopy images of the *in vitro* culture. After D8, the formation of macrophages is complete.

Table S.1: Clinical data of three CLL patients.

**Patient**	**P1**	**P2**	**P3**
**Mutated/non-mutated**	IGH-mutated (M-CLL)	IGH mutated (M-CLL)<br>TP53 non-mutated	IGH non-mutated (U-CLL)<br>TP53 non-mutated
**Treatment naïve or BTK/BCL2 inhibitor**	1st line: 6 cycles of RCD in 2012<br>6 cycles of R-Bendamustine starting early 2018→ Aug 2018.<br><br>Treated with weekly 7.5 mg MTX for skin lesions, from Jan → May 2022	No information	1st line: Ibrutinib from Aug 2017 → Jan 2018.<br>2nd line: 6 cycles of RCD starting Aug 2018.<br>3rd line: 6 cycles of R-Bendamustine starting Jul 2021.<br>4th line: R-Venetoclax (Bcl-2 inhibitor) started late Sep 2022, anti-CD20 started early Oct 2022).<br>6th cycle of Rituximab on Mar 27, 2023
**Copy number alterations and genetic drivers**	Not defined<br>(no cytogenetic test)	Absence of clonal chromosomal anomaly<br>Absence of ATM and TP53 deletions	17p deletions without TP53 mutations<br>Absence of ATM deletion
**Age**	76	66	83
**Sex**	M	M	M
**Sample**	Frozen	Frozen	Fresh

Principal Component Analysis results

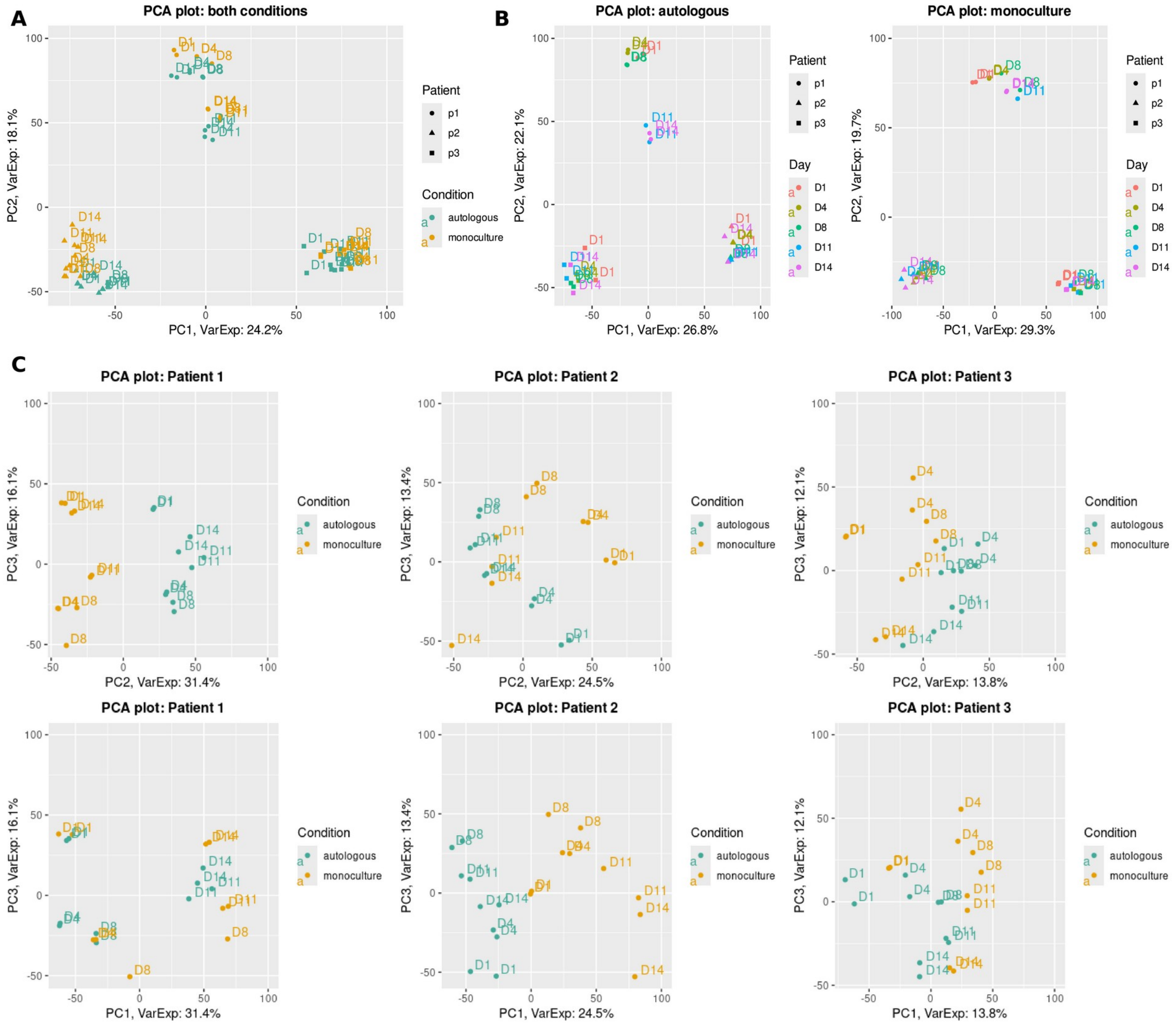

Fig. S.3: Principal Component Analysis of autologous and monoculture, showing the high heterogeneity between patients.

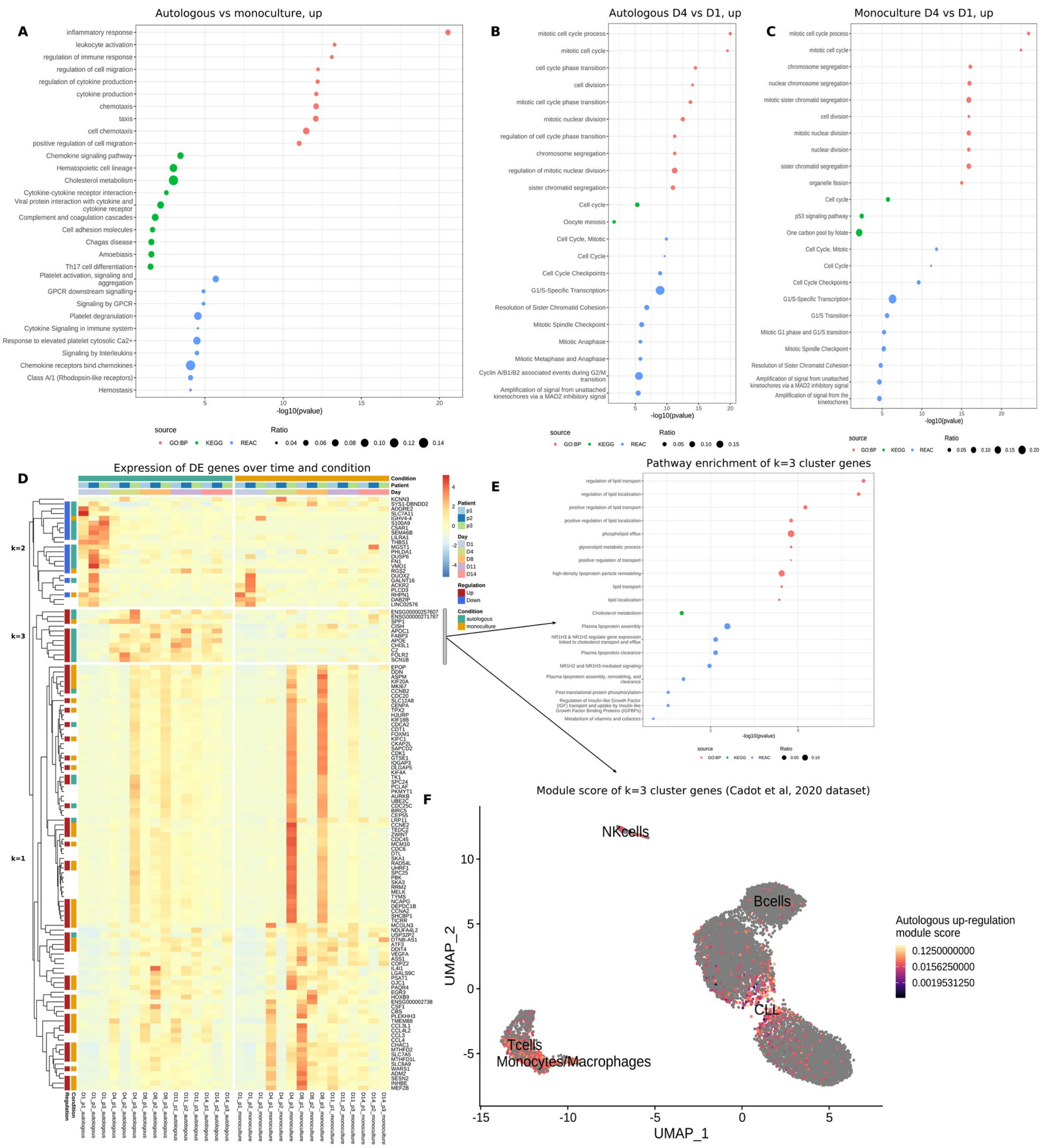

Fig. S.4: A Pathway enrichment analysis of the up-regulated genes in the autologous vs monoculture. **B**, **C** Pathway enrichment analysis of the up-regulated genes in D4 vs D1 comparison in the autologous and monoculture, respectively. **D** Expression of the differentially expressed genes in autologous and monoculture in t vs $t-1$ comparison. **E** Pathway enrichment analysis of the $k=3$ genes. **F** UMAP of CITE-seq dataset of CLL PBMC from Cadot *et al*. and score of the $k=3$ gene module. All the necessary scripts to reproduce the results can be found in the GitHub repository.

Ligand-receptor analysis

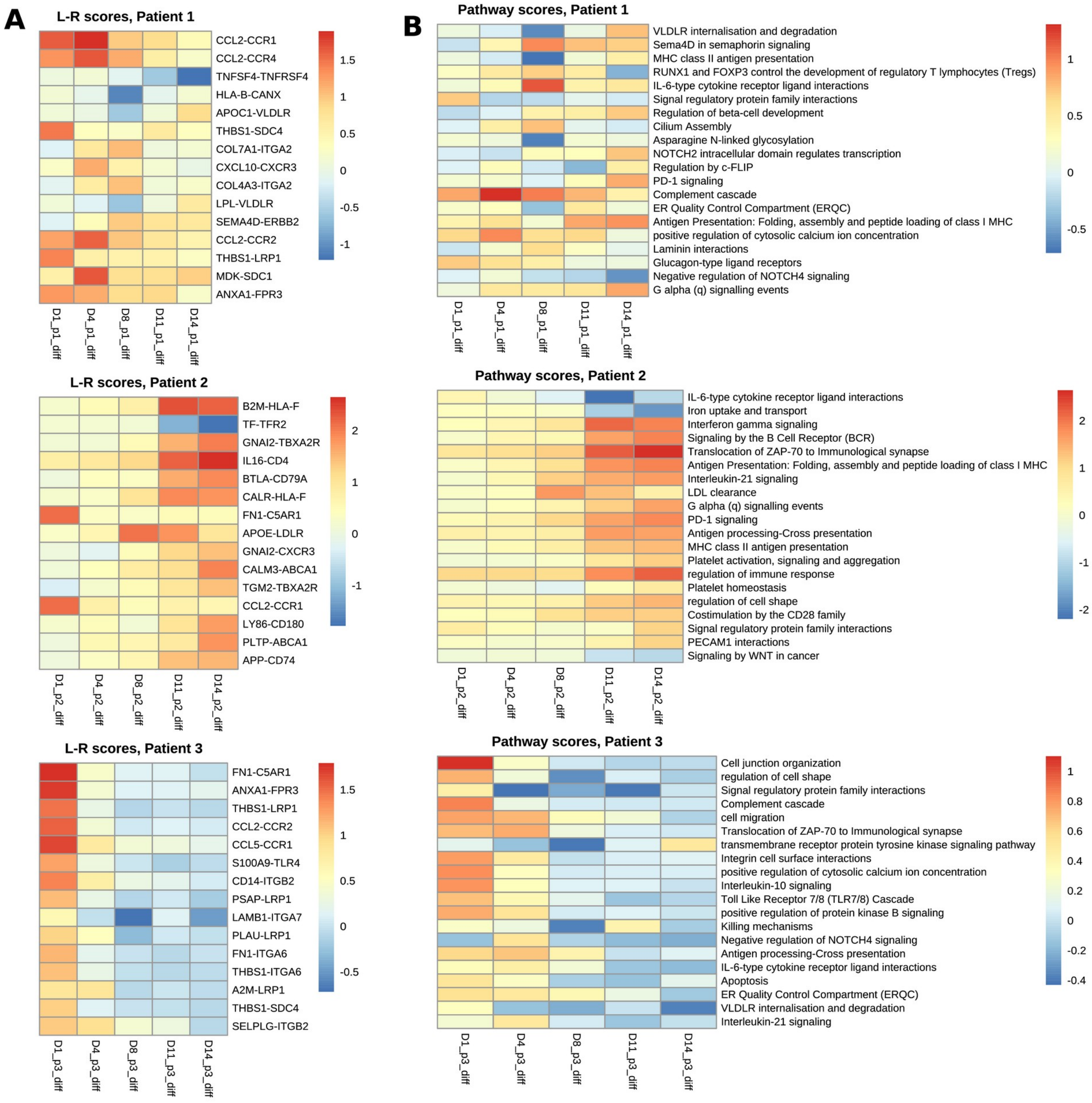

Fig. S.5: A Ligand-receptor pair activation. **B** Pathway scores for the expressed L-R pairs.

Independent Component Analysis (ICA)

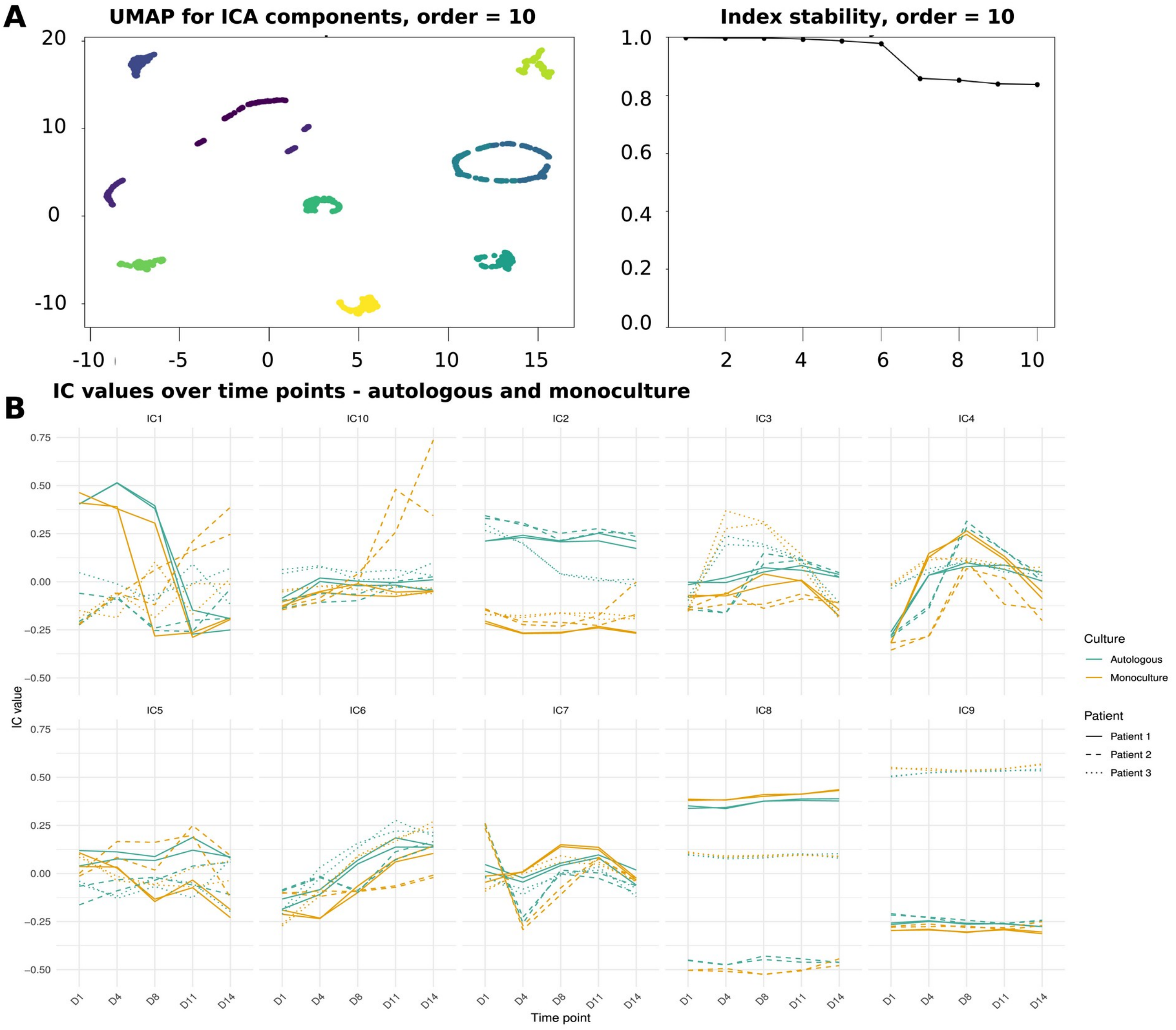

Fig. S.6: A Index stability - autologous and monoculture. **B** Heatmap of the metasample matrix from ICA. **C** ICA - TF activity score.

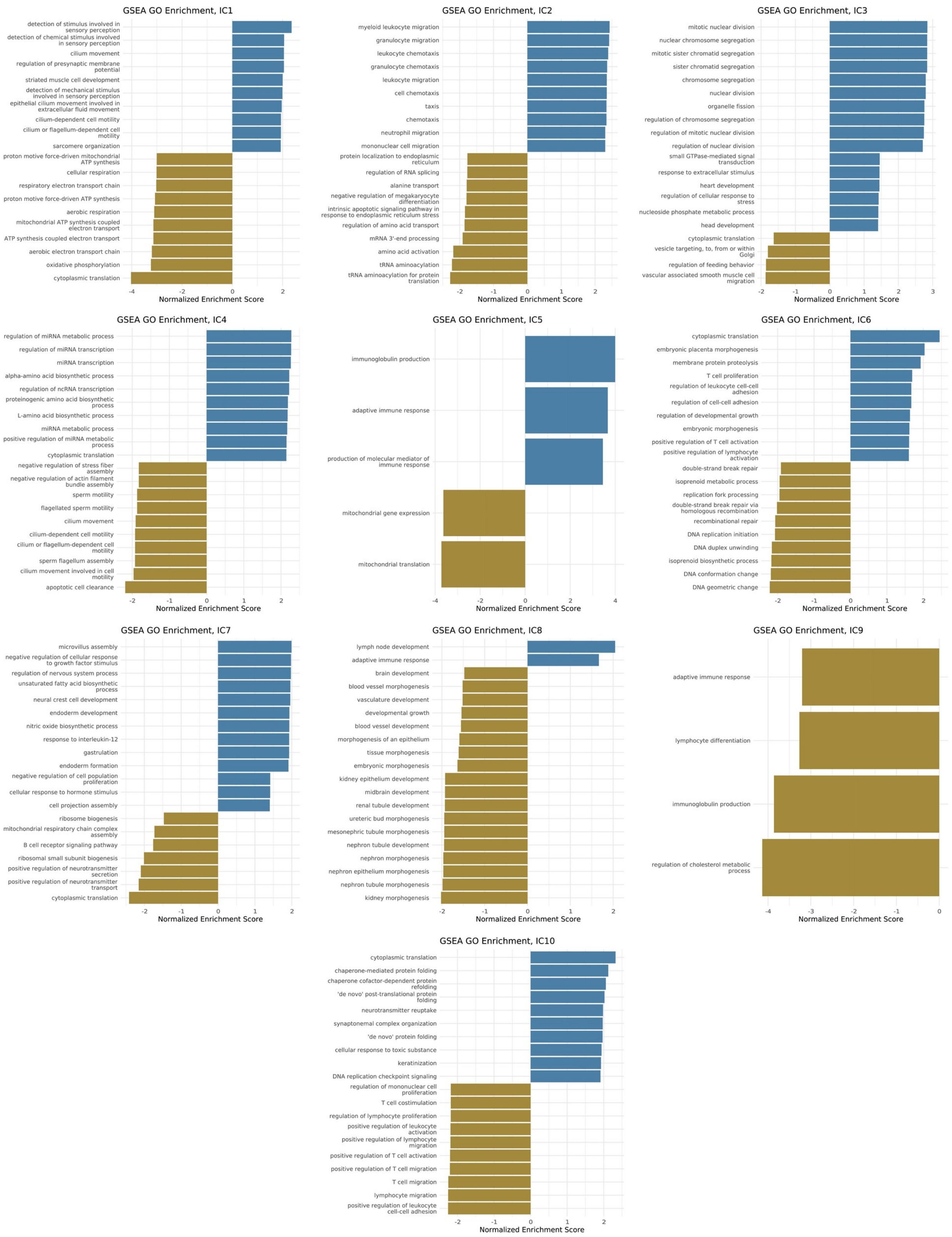

Fig. S.7: Gene set enrichment analysis on the independent components.

Network analysis

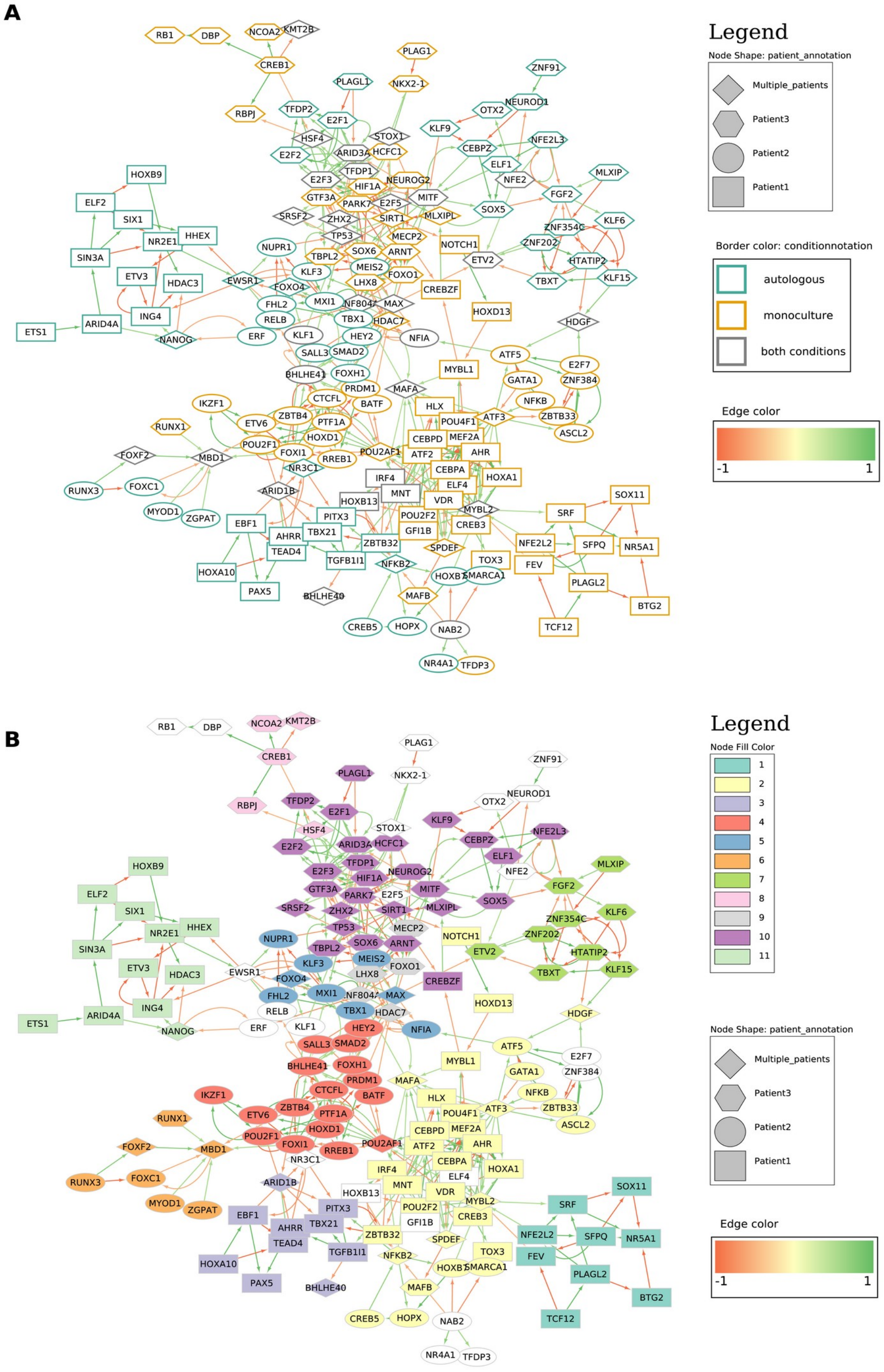

Fig. S.8: A TF-TF network with nodes coloured by condition. **B** TF-TF network with nodes coloured by module.

Validation of TF selection through external dataset analysis

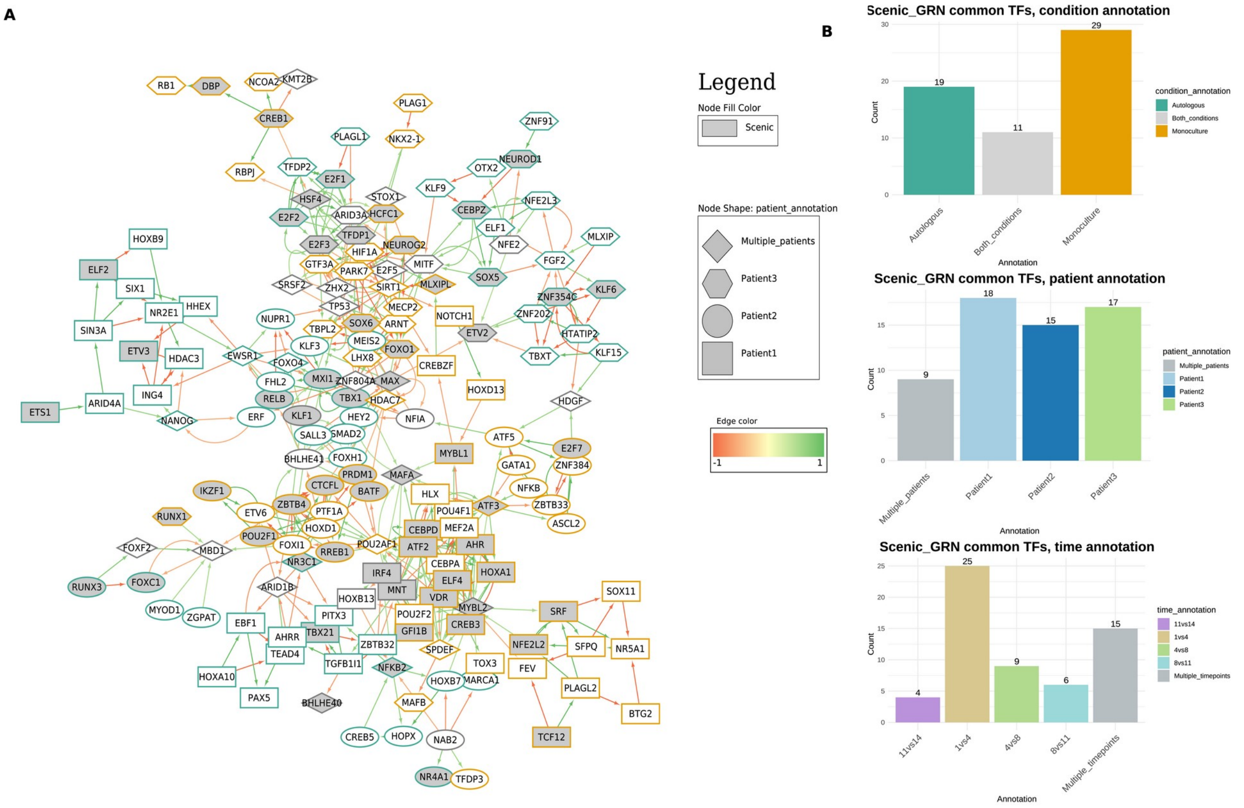

Fig. S.9: A Mapping of the overlapping TFs onto our inferred GRN. **B** Partition of the overlapping TFs according to condition, patient and time-point. All the necessary scripts to reproduce the results can be found in the GitHub repository.

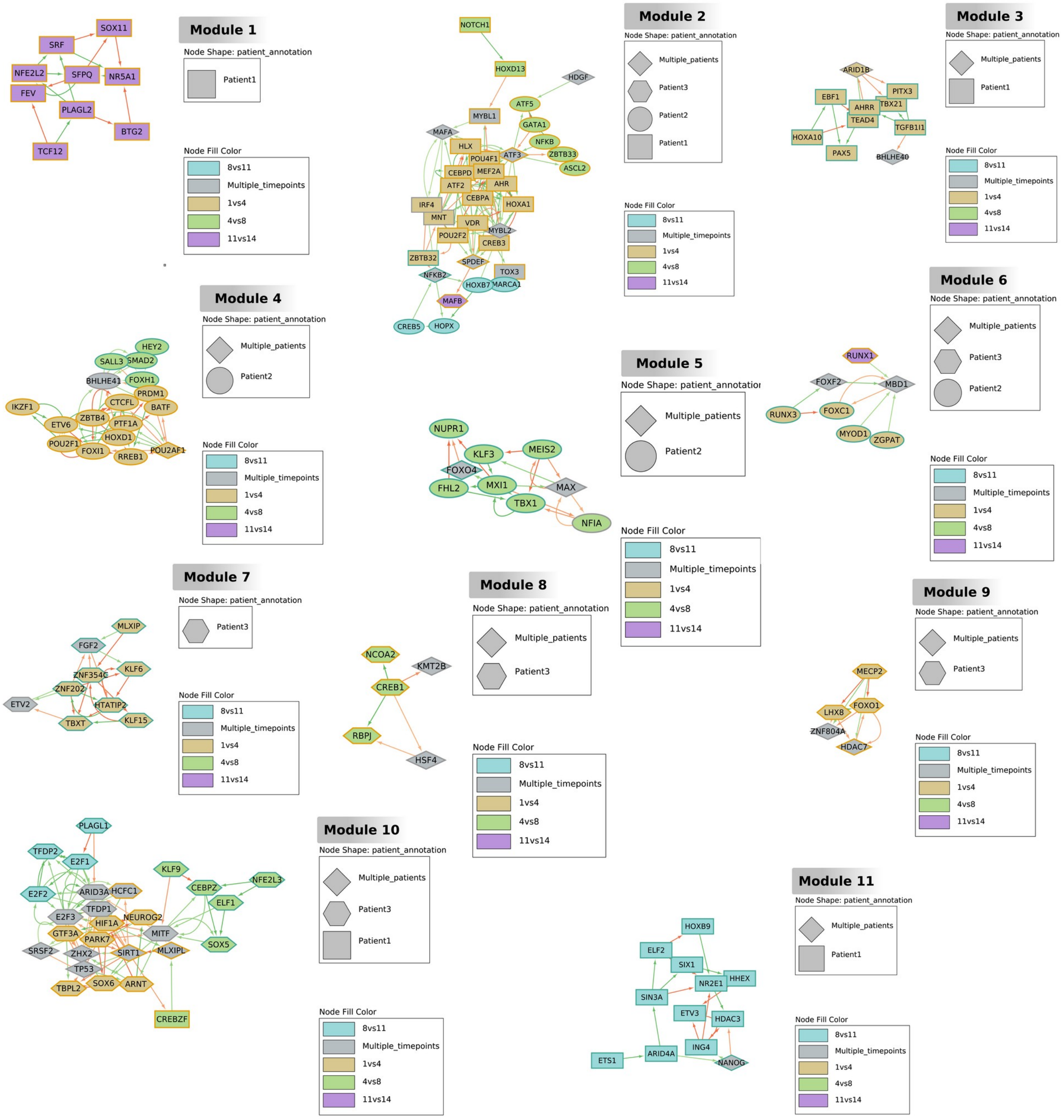

Fig. S.10: Annotation of modules with nodes' features.

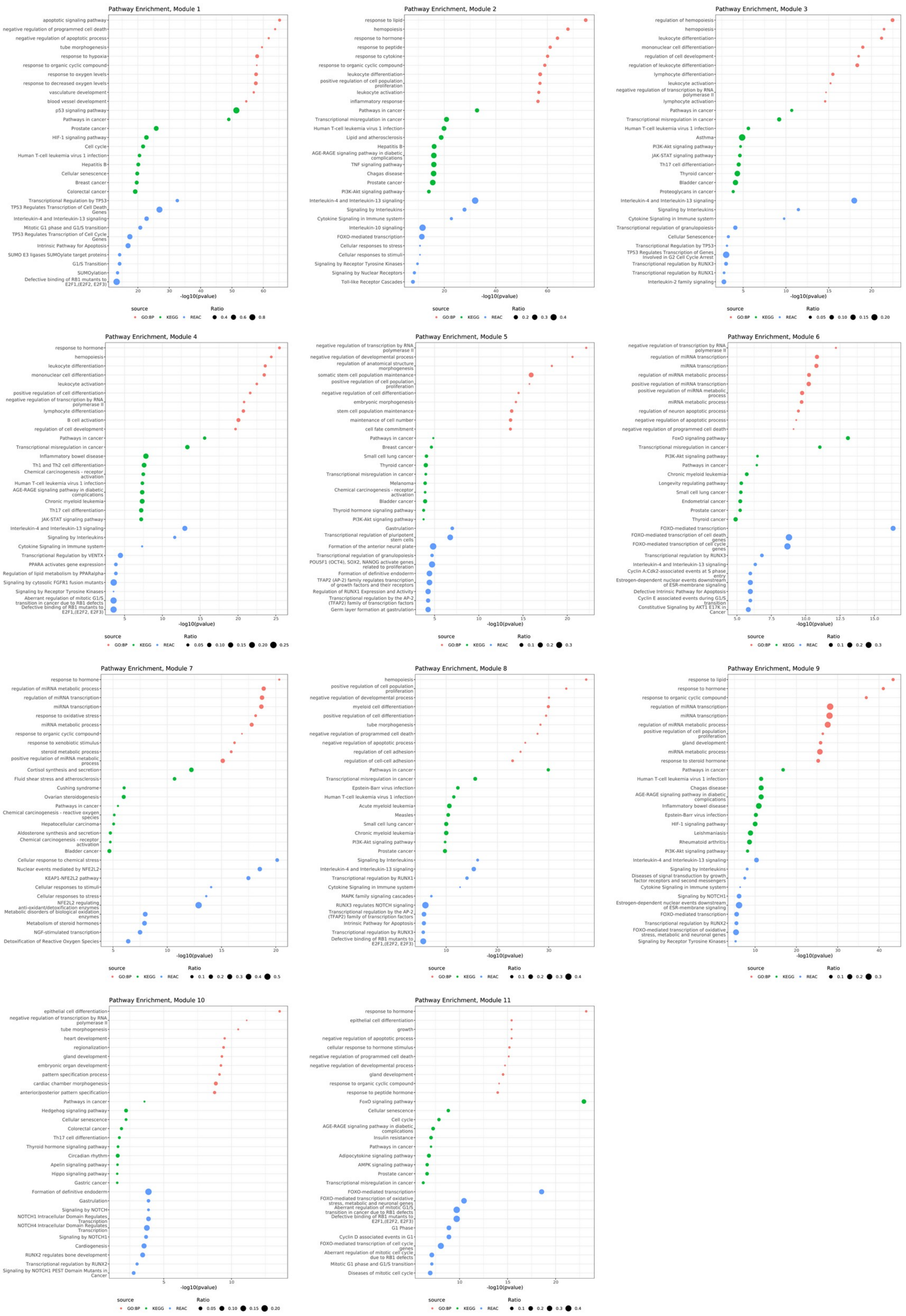

Fig. S.11: Pathway enrichment analysis of each cluster of TFs (including their targets).
